# Activation of the M3AChR and Notch1/HSF1 Signaling Pathway by Choline Alleviates Angiotensin II-Induced Cardiomyocyte Apoptosis

**DOI:** 10.1155/2021/9979706

**Published:** 2021-08-30

**Authors:** Man Xu, Xue-Yuan Bi, Xiao-Rong Xue, Xing-Zhu Lu, Qiong-Ge Li, Qiang Jian, Jian-Yong Sun

**Affiliations:** ^1^Department of Clinical Pharmacy, Xi'an People's Hospital (Xi'an Fourth Hospital), Xi'an, 710004 Shaanxi Province, China; ^2^Department of Pharmacy, Hong Hui Hospital, Xi'an Jiaotong University, Xi'an, 710054 Shaanxi Province, China; ^3^Department of Pharmacy, Second Affiliated Hospital of Xi'an Jiaotong University Medical School, No.157, West Fifth Road, Xi'an, Shaanxi 710004, China; ^4^Department of Thoracic Surgery, Tangdu Hospital, Air Force Medical University, Xi'an, 710004 Shaanxi Province, China

## Abstract

Angiotensin II- (Ang II-) induced cardiac hypertrophy and apoptosis are major characteristics of early-stage heart failure. Choline exerts cardioprotective effects; however, its effects on Ang II-induced cardiomyocyte apoptosis are unclear. In this study, the role and underlying mechanism of choline in regulating Ang II-induced cardiomyocyte apoptosis were investigated using a model of cardiomyocyte apoptosis, which was induced by exposing neonatal rat cardiomyocytes to Ang II (10^−6^ M, 48 h). Choline promoted heat shock transcription factor 1 (HSF1) nuclear translocation and the intracellular domain of Notch1 (NICD) expression. Consequently, choline attenuated Ang II-induced increases in mitochondrial reactive oxygen species (mtROS) and promotion of proapoptotic protein release from mitochondria, including cytochrome *c*, Omi/high-temperature requirement protein A2, and second mitochondrial activator of caspases/direct inhibitor of apoptosis-binding protein with low P. The reversion of these events attenuated Ang II-induced increases in cardiomyocyte size and numbers of terminal deoxynucleotidyl transferase deoxyuridine triphosphate nick end labeling-positive cells, presumably via type 3 muscarinic acetylcholine receptor (M3AChR). Indeed, downregulation of M3AChR or Notch1 blocked choline-mediated upregulation of NICD and nuclear HSF1 expression, as well as inhibited mitochondrial apoptosis pathway and cardiomyocyte apoptosis, indicating that M3AChR and Notch1/HSF1 activation confer the protective effects of choline. *In vivo* studies were performed in parallel, in which rats were infused with Ang II for 4 weeks to induce cardiac apoptosis. The results showed that choline alleviated cardiac remodeling and apoptosis of Ang II-infused rats in a manner related to activation of the Notch1/HSF1 pathway, consistent with the *in vitro* findings. Taken together, our results reveal that choline impedes oxidative damage and cardiomyocyte apoptosis by activating M3AChR and Notch1/HSF1 antioxidant signaling, and suggest a novel role for the Notch1/HSF1 signaling pathway in the modulation of cardiomyocyte apoptosis.

## 1. Introduction

Apoptosis is implicated in various cardiovascular disorders, including myocardial infarction and heart failure (HF) [1], in which the activation of apoptotic pathways may contribute to cardiomyocyte loss and subsequent cardiac dysfunction. HF is linked to hyperactivation of the renin-angiotensin-aldosterone system; this hyperactivation, mediated by angiotensin II (Ang II), is thought to trigger cardiomyocyte apoptosis [2, 3]. However, the mechanisms underlying Ang II-induced cardiomyocyte apoptosis are unclear. Thus, it is critical to investigate the mechanisms associated with cardiomyocyte apoptosis and to develop novel therapies to prevent and treat HF.

The mitochondrial death pathway, also called the intrinsic cell death pathway, induces cell death in response to intracellular stressors such as increased oxidative stress, serum deprivation, or deoxyribonucleic acid (DNA) damage [1]. This pathway is regulated by antiapoptotic B-cell lymphoma- (Bcl-) 2 proteins [4]. Bcl-2 proteins suppress apoptosis by inhibiting the proapoptotic Bcl-2 family proteins Bax and Bak. Under stressful conditions, activated Bax/Bak permeabilizes the outer mitochondrial membrane, allowing the release of apoptogenic factors, including cytochrome *c*, Omi/high-temperature requirement protein A2 (Omi/HtrA2), and the second mitochondrial activator of caspases/direct inhibitor of apoptosis-binding protein with low P (Smac/DIABLO) into the cytosol, where they subsequently activate the caspase cascade [5]. Previous studies have suggested that the mitochondrial death pathway is involved in cardiovascular diseases that share mitochondrial dysfunction as a common pathogenetic mechanism, including myocardial ischemia/reperfusion (I/R) injury [6] and cardiac remodeling [7]. Regardless, the contribution of the mitochondrial death pathway to Ang II-induced cardiomyocyte apoptosis is unknown.

Mitochondrial reactive oxygen species (mtROS) generation during cellular aerobic respiration and metabolism is implicated in cardiovascular pathologies [8]. The production of large quantities of mtROS via Ang II activation can trigger the destruction of intracellular proteins, organelles, and DNA [9]. Attenuating mtROS levels and thereby reducing oxidative stress can alleviate cardiomyocyte apoptosis. Preventing oxidative stress is necessary to maintain cardiomyocyte homeostasis. The Notch1 protein can act as an endogenous cardioprotective factor by reducing myocardial reactive oxygen species (ROS) production, inhibiting the opening of mitochondrial permeability transition pores, alleviating cardiomyocyte apoptosis, and reducing the myocardium infarct size [10]. Heat shock transcription factor 1 (HSF1) is involved in the cellular response to divergent pathophysiological stress, such as myocardial I/R [11, 12]. In unstressed cells, HSF1 is present in both the cytoplasm and nucleus, while, in response to stress, HSF1 accumulates within the nucleus. Notably, HSF1 induction inhibits ROS production, as evidenced by the reduced expression of 4-hydroxynonenal, a key oxidative stress marker, in ischemic livers. Notch1 signaling was recently reported to promote HSF1 activity, which in turn inhibits the nucleotide-binding domain, leucine-rich repeat family pyrin domain containing 3 (NLRP3) function, and hepatocellular apoptosis, consequently alleviating I/R-induced liver injury [13]. Nonetheless, the direct role of Notch/HSF1 in mtROS generation and the intrinsic cell death pathway during cardiomyocyte apoptosis has not been confirmed. Delineating this link would help to identify novel therapeutic targets and pharmacological regimens to prevent or alleviate myocardial injuries.

Choline, a type 3 muscarinic acetylcholine receptor (M3AChR) agonist, is a precursor in the biosynthesis of acetylcholine, a vagal neurotransmitter. M3AChR activation by choline confers protective effects against various cardiac diseases, including arrhythmias, ischemic myocardial injuries, and I/R injury [14]. Choline has been reported to alleviate cardiac hypertrophy by regulating metabolic remodeling and the mitochondrial unfolded protein response [15]. However, no previous studies have examined the effects of choline on cardiomyocyte apoptosis. Therefore, in this study, the role and molecular mechanisms of choline in the regulation of Ang II-induced cardiomyocyte apoptosis were investigated. It is also unclear whether Notch1/HSF1 signaling is involved in the cardioprotective effects of choline. Accordingly, the effects of choline on cardiomyocyte apoptosis in rats and neonatal rat ventricular myocytes (NRVMs) treated with Ang II were also investigated. Our results will provide mechanistic insights into therapeutic strategies to attenuate cardiomyocyte apoptosis in cardiovascular diseases.

## 2. Materials and Methods

### 2.1. NRVM Isolation and Culturing

The experimental procedures were performed in accordance with the Guidelines on the Care and Use of Laboratory Animals and were approved by the Ethics Committee of Xi'an People's Hospital (Xi'an Fourth Hospital). NRVMs were prepared as previously described [16]. Briefly, the ventricles of 1- to 3-day-old neonatal Sprague Dawley rats were digested with 0.1% collagenase for 20 min. Cardiomyocytes were collected based on their differential adhesion and seeded at a density of 5 × 10^4^ cells/well in six-well plates. The cells were grown in low-glucose Dulbecco's modified Eagle's medium (L-DMEM) containing 10% fetal bovine serum, penicillin (50 U/mL), and streptomycin (50 *μ*g/mL) at 37°C in humidified air containing 5% CO_2_. The culture medium was renewed every other day. The experimental groups were as follows: (1) CTR (control), cultured in L-DMEM; (2) Ang II group, incubated with Ang II (1 *μ*mol/L); (3) Ang II+choline group, incubated with choline (0.5 mmol/L) and Ang II (1 *μ*mol/L); and (4) Ang II+choline+4-DAMP group, incubated with 4-DAMP (4-diphenylacetoxy-N-methylpiperidine methiodide, a selective M3AChR antagonist, 1 *μ*mol/L), choline, and Ang II. Choline was added 30 min before Ang II, while 4-DAMP was added 30 min before choline. The chosen concentrations of Ang II [17], choline [15], and 4-DAMP [18] were based on preliminary data and previously published reports.

### 2.2. Terminal Deoxynucleotidyl Transferase Deoxyuridine Triphosphate Nick End Labeling (TUNEL) Staining

NRVM apoptosis was assessed using a DeadEnd Fluorometric TUNEL system (Promega, Madison, WI, USA). Briefly, cells were seeded onto collagen-coated coverslips and fixed with 4% paraformaldehyde (Sigma), after which the TUNEL reaction mixture was added. Nuclei were stained using 4′,6-diamidino-2-phenylindole (DAPI). The cells were imaged using a fluorescence microscope (TE-2000U; Nikon, Japan). In each group, apoptotic cells in 10 randomly chosen fields were counted to calculate the apoptotic index as follows:

(1) $$\begin{array}{c} \text{Apoptotic index}=\text{positive cells}/\left(\text{positive cells}+\text{negative cells}\right)\times 100\%. \end{array}$$

### 2.3. mtROS Detection

mtROS was detected using MitoSOX Red (Invitrogen, Carlsbad, CA, USA). Briefly, cells were incubated with 5 *μ*mol/L MitoSOX Red for 30 min at 37°C in darkness and washed twice with phosphate-buffered saline (PBS) for 10 min per wash. After fixation for 15 min in 4% paraformaldehyde in PBS, the cells were again washed twice with PBS for 10 min per wash. Then, the cells were incubated with MitoTracker Green (200 nM, Beyotime Biotechnology, Beijing, China) for 20 min at 37°C in darkness to label the mitochondria, after which they were washed a final time with PBS for 10 min. Fluorescent images were acquired using a confocal microscope (Nikon C2, Nikon, Tokyo, Japan).

### 2.4. Isolation of Cytosolic and Mitochondrial Fractions

NRVMs were washed with PBS and collected via centrifugation at 1000 rpm for 5 min at room temperature. The supernatant was subsequently harvested for mitochondrial and cytosolic isolation using a cell mitochondria isolation kit. Briefly, cells were gently rinsed with prechilled PBS and then centrifuged at 600 × g for 5 min at 4°C. A mitochondrial isolation reagent was added to the precipitate. The lysate was transferred to a glass homogenizer, stirred by forcefully passing the cells, and centrifuged at 600 × g for 10 min at 4°C. The supernatant was further centrifuged at 11,000 × g for 10 min at 4°C, and the mitochondria were collected from the precipitate. The remaining supernatant was centrifuged at 12,000 × g for 10 min at 4°C, and the supernatant was collected as the cytosolic protein fraction. Protein concentrations were quantified using the NanoPhotometer P-330 (Implen, Germany).

### 2.5. Isolation of Cytosolic and Nuclear Fractions

Nuclear and cytoplasmic extracts were prepared using an NE-PER Nuclear and Cytoplasmic Extraction Reagent kit (ThermoFisher Scientific, Waltham, MA, USA).

### 2.6. Western Blot Analysis

Samples containing 30 g protein were separated using 10% sodium dodecyl sulphate-polyacrylamide gel electrophoresis. After wet transfer onto polyvinylidene difluoride membranes (Merck Millipore, Billerica, MA, USA) at 300 mA for 1.5 h, the membranes were incubated for 1 h at ambient temperature with a blocking solution of Tris-buffered saline containing 0.1% Tween (TBST) and 5% skim milk. Afterward, the membranes were incubated overnight with the primary antibody at 4°C with gentle rocking. The membranes were then washed five times in TBST for 5 min per wash and incubated with the appropriate horseradish peroxidase-conjugated secondary antibody for 1 h at room temperature. Subsequently, the membranes were washed five times in TBST for 5 min per wash and developed with SuperSignal-blotting detection reagents (Millipore).

The primary antibodies used in the experiments were as follows: Bcl-2 (1 : 2000; Cell Signaling Technology), Bax (1 : 1000; Cell Signaling Technology), Omi/HtrA2 (1 : 1000; Cell Signaling Technology), Smac/DIABLO (1 : 1000; Cell Signaling Technology), cytochrome *c* (1 : 1000; Cell Signaling Technology), Notch1 intracellular domain (NICD, 1 : 2000; ThermoFisher Scientific), HSF1 (1 : 1000; Thermo Fisher Scientific), and cleaved-caspase-3 (1 : 1000; Cell Signaling Technology). Equal protein loading was determined by western blotting using a mouse GAPDH antibody as a total protein-loading control (mouse polyclonal, 1 : 5000; Sigma-Aldrich, St. Louis, MO, USA). Anti-Lamin B1 antibody (1 : 2000; Abcam) was used as a loading control for the cell nucleus. Anti-COX IV antibody was used as a loading control for the mitochondria. The band intensities were analyzed using the Gel-Pro Analyzer (Media Cybernetics, Bethesda, MD, USA).

### 2.7. Small Interfering Ribonucleic Acids (siRNAs)

NRVMs were transiently transfected with M3AChR and Notch1 small interfering RNA (siRNA) or negative control (NC) siRNA (GenePharma, Shanghai, China) using Lipofectamine 2000 transfection reagent (Invitrogen, Carlsbad, CA, USA) according to the manufacturer's instructions.

### 2.8. Immunofluorescence Staining

Cultured NRVMs were washed three times with PBS, fixed in 4% paraformaldehyde for 30 min, permeabilized with 0.3% Triton X-100 for 1 h, and then blocked for 1 h in PBS with goat serum. Next, the cells were incubated with primary antibodies against HSF1 (1 : 100; ThermoFisher Scientific) or Notch1 (1 : 100; ThermoFisher Scientific) overnight at 4°C. Afterward, the sections were incubated with goat anti-rabbit immunoglobulin G (heavy + light chains) superclonal secondary antibody or goat anti-mouse immunoglobulin G secondary antibody (Beyotime) for 1 h. Cell nuclei were counterstained with DAPI (Beyotime). The sections were imaged using a laser scanning confocal microscope (Nikon, Tokyo, Japan) and analyzed with ImageJ.

### 2.9. Animals and Manipulations

Adult male Sprague-Dawley rats (8–10 weeks old) were supplied by the Experimental Animal Centre of Xi'an Jiaotong University. After a week of adaptation, rats were randomly divided into sham, Ang II, and Ang II+choline groups (*n* = 8 in each). For all three groups, rats were anesthetized with ether, and an Alzet osmotic minipump (model 1003D or 2001, Alza Corp) containing Ang II dissolved in saline or saline alone (sham group) was implanted subcutaneously in accordance with the manufacturer's protocol. After infusion with saline or Ang II at a dose of 400 ng/kg/min [19], rats in the Ang II+choline group received intraperitoneal injection of choline (7 mg/kg/day) every day, whereas rats in the sham and Ang II groups received equal volumes of saline solution. All rats were infused and injected for 4 weeks. At the end of the study, the rats were euthanized with sodium pentobarbital (100 mg/kg, intraperitoneally), and the left ventricles of the heart were collected for further experiments.

### 2.10. Histological and Morphological Analyses

Rat hearts from each group were excised, washed, fixed in 4% formalin, dehydrated through graded alcohols, and embedded in paraffin wax. Then, blocks were cut into 2 *μ*m thick paraffin sections, deparaffinized by immersing in xylene, and rehydrated. Subsequently, the tissue sections were stained with hematoxylin and eosin (H&E) and Masson's trichrome. The myocyte cross-sectional diameter and the area of fibrosis were measured using Image-Pro Plus v.6.0 (Media Cybernetics, Silver Spring, MD, USA).

### 2.11. Statistical Analysis

The data are presented as means ± standard deviation (SD). Statistical analyses were conducted using one-way analysis of variance followed by Tukey's multiple comparison test. For all analyses, *P* < 0.05 indicates statistical significance.

## 3. Results

### 3.1. Ang II Treatment Reduced the Bcl-2/Bax Ratio and Increased the Expression of Cleaved-Caspase-3 in NRVMs

Ang II, the primary effector molecule of the renin-angiotensin-aldosterone system, induces cardiomyocyte apoptosis. The downregulation of antiapoptosis proteins, including Bcl-2, and the upregulation of proapoptosis proteins, such as Bax, are hallmarks of cardiomyocyte apoptosis. Perturbations in the Bcl-2/Bax ratio following an apoptotic stimulus may promote cell death [20].

Changes in Bcl-2, Bax, and cleaved-caspase-3 expression following Ang II administration were tracked over time. NRVMs were exposed to Ang II (10^−6^ mol/L) for 12, 24, 36, 48, or 60 h. Ang II time-dependently reduced the Bcl-2/Bax ratio and increased cleaved-caspase-3 expression, with maximal changes at 48 h (Supplementary material online, Figure S1A and B). Next, NRVMs were treated with Ang II (10^−8^ to 10^−4^ mol/L) for 48 h. Ang II decreased the Bcl-2/Bax ratio and enhanced cleaved-caspase-3 expression in a dose-dependent manner, with peak effects observed in cells treated with a dose of 10^−6^ mol/L (Supplementary material online, Figure S1C and D). Therefore, Ang II administration at 10^−6^ mol/L for 48 h was used in subsequent experiments.

### 3.2. Choline Inhibited Ang II-Induced Cardiomyocyte Apoptosis via M3AChR Activation

**Figure 1:**
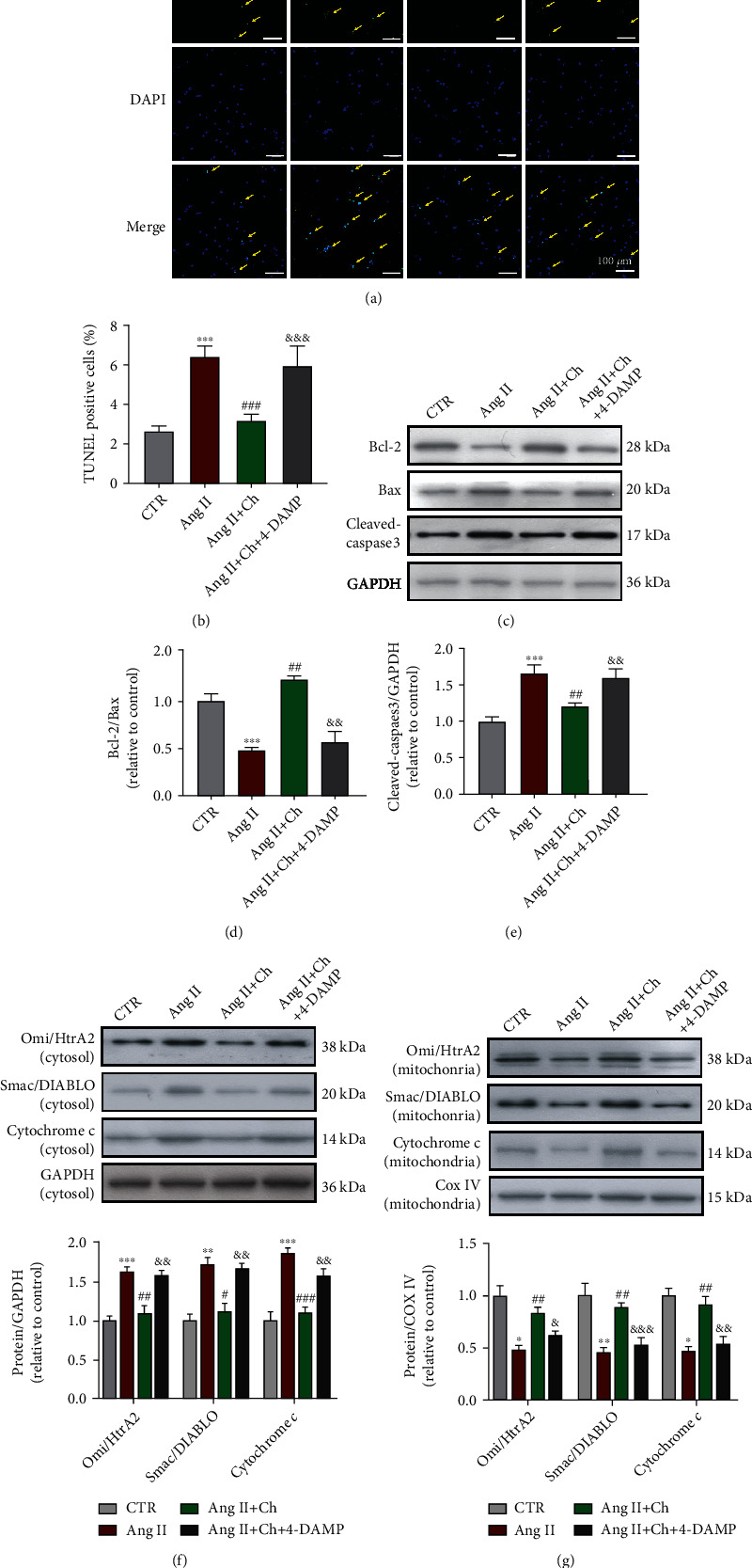
Choline attenuated angiotensin II- (Ang II) induced stimulation of the mitochondrial death pathway and cardiomyocyte apoptosis. (a) 4-Diphenylacetoxy-N-methylpiperidine methiodide (4-DAMP) administration blocked the effect of choline on terminal deoxynucleotidyl transferase deoxyuridine triphosphate nick end labeling- (TUNEL-) positive neonatal rat ventricular myocytes (NRVMs). Scale bar: 50 *μ*m. (b) The ratio of TUNEL-positive cells to total cells. Data are presented as means ± SD (*n* = 5 independent experiments). (c) Choline treatment increased the Bcl-2/Bax ratio and inhibited caspase-3 activation. The protective effects of choline were reversed by 4-DAMP. (d, e) Quantification of Bcl-2/Bax and cleaved-caspase-3. Data are presented as means ± SD (*n* = 5 independent experiments). (f) Representative image and graph showing levels of Omi/high-temperature requirement protein A2 (HtrA2), the second mitochondrial activator of caspases/direct inhibitor of apoptosis-binding protein with low P (Smac/DIABLO), and cytochrome *c* in the cytosol fraction. Data are presented as means ± SD (*n* = 5 independent experiments). (g) Representative western blot images and data of Omi/HtrA2, Smac/DIABLO, and cytochrome *c* in the mitochondrial fraction. Data are presented as means ± SD (*n* = 5 independent experiments). ^∗^*P* < 0.05, ^∗∗^*P* < 0.01, and ^∗∗∗^*P* < 0.001 versus control (CTR); ^#^*P* < 0.05, ^##^*P* < 0.01, and ^###^*P* < 0.001 versus Ang II; ^&^*P* < 0.05, ^&&^*P* < 0.01, and ^&&&^*P* < 0.001 versus the choline-treated Ang II group.

A TUNEL assay was performed on Ang II-treated NRVMs. As shown in Figures 1(a) and 1(b), choline treatment significantly reduced the number of Ang II-induced TUNEL-positive NRVMs. This effect was reversed by treatment with the selective M3AChR antagonist 1,1-dimethyl-4-diphenylacetoxypiperidinium iodide (4-DAMP) (10^−6^ mol/L). As shown in Figures 1(c) and 1(d), choline increased the expression of the antiapoptotic factor Bcl-2 and increased the Bcl-2/Bax ratio in Ang II-treated NRVMs. Choline administration reduced cleaved-caspase-3 expression (Figure 1(e)). The effects of choline were reversed by 4-DAMP.

The release of cytochrome *c*, Omi/HtrA2, and Smac/DIABLO from the mitochondria under different conditions was quantified. In the control group, low levels of cytochrome *c*, Omi/HtrA2, and Smac/DIABLO were released from the mitochondria into the cytosol. After Ang II treatment, their levels were significantly increased in the cytosol and decreased in the mitochondria. Choline administration inhibited the release of cytochrome *c*, Omi/HtrA2, and Smac/DIABLO into the cytosol. The effects of choline were in turn reversed by the selective M3AChR antagonist 4-DAMP (Figures 1(f) and 1(g)).

### 3.3. Choline Attenuated Ang II-Induced Increases in mtROS Levels and Cell Surface Area

**Figure 2:**
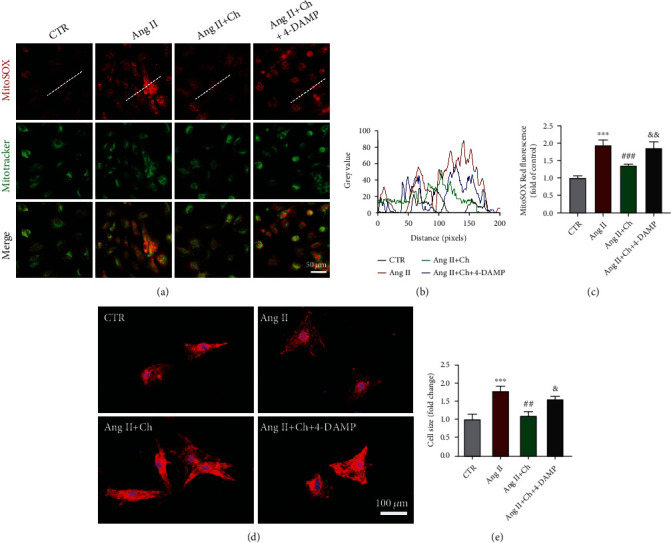
Choline counteracted the Ang II-induced increase in mitochondrial reactive oxygen species (mtROS) levels and surface area. (a–c) Representative images and data showing mtROS levels. MitoSOX Red fluorescence colocalized with MitoTracker Green. Data are presented as means ± SD (*n* = 4 independent experiments). Scale bar: 50 *μ*m. (d) Representative immunofluorescence staining of NRVMs with *α*-actinin antibodies (red); the nuclei were stained with 4′,6-diamidino-2-phenylindole (DAPI; blue). Scale bar: 100 *μ*m. (e) Statistical analysis of the cardiomyocyte surface area. *n* = 5 independent experiments. Data are presented as means ± SD. ^∗∗∗^*P* < 0.001 versus control (CTR); ^##^*P* < 0.01 and ^###^*P* < 0.001 versus Ang II; ^&^*P* < 0.05 and ^&&^*P* < 0.01 versus the choline-treated Ang II group.

ROS are highly reactive molecules, mainly generated inside mitochondria that can oxidize DNA, proteins, and lipids. At physiological levels, mtROS function as “redox messengers” in intracellular signaling and regulation, whereas overproduction of mtROS leads to injury of the cell membrane integrity causing altered permeability, change in protein expression, and DNA damage [21, 22]. It has been already established that mtROS plays a vital role in mediating apoptosis through the intrinsic pathway [23]. In this study, the fluorescent probe MitoSOX Red was used to detect changes in mtROS levels. Ang II significantly elevated mtROS levels, which was then reversed by choline (Figures 2(a)–2(c)). In turn, the protective effect of choline was reversed by 4-DAMP. Changes in cell surface area during Ang II stimulation were also investigated. Choline markedly attenuated Ang II-induced increases in cell surface area, which was then reversed by 4-DAMP (Figures 2(d) and 2(e)).

### 3.4. Choline Increased Notch1 Intracellular Domain (NICD) Expression and Potentiated HSF1 Translocation in Ang II-Treated NRVMs

The Notch1 signaling pathway plays essential roles in cell communication. After binding to the ligand Jagged/Delta, Notch1 releases its intracellular domain, NICD, which then enters the nucleus and binds to transcription factors, resulting in the transcription of downstream signaling transduction molecules [24, 25].

**Figure 3:**
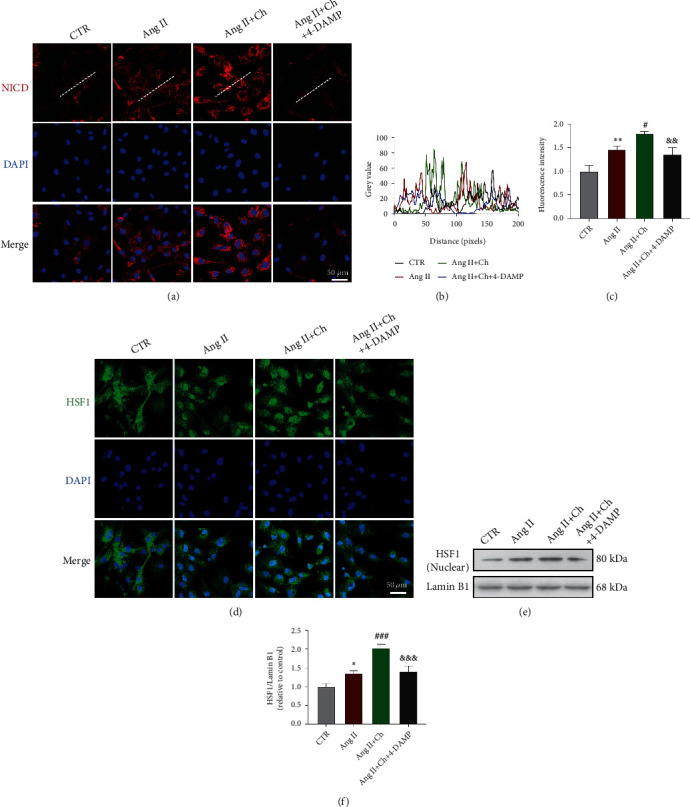
Choline improved Notch1 intracellular domain (NICD) expression and promoted nuclear translocation of heat shock transcription factor 1 (HSF1). (a–c) NICD protein levels (red) in NRVMs were quantified via immunostaining with the indicated antibodies; nuclei were stained with DAPI (blue). Scale bar: 50 *μ*m. Data are presented as means ± SD; *n* = 5 independent experiments. (d) HSF1 immunofluorescence staining. Scale bar: 50 *μ*m. *n* = 5 independent experiments. (e, f) Representative images and graph of HSF1 protein levels in the nuclear fraction. Data are presented as means ± SD (*n* = 5 independent experiments). ^∗^*P* < 0.05 and ^∗∗^*P* < 0.01 versus control (CTR); ^#^*P* < 0.05 and ^###^*P* < 0.001 versus Ang II; ^&&^*P* < 0.01 and ^&&&^*P* < 0.001 versus the choline-treated Ang II group.

Immunofluorescence and confocal microscopy showed that choline significantly increased NICD levels in Ang II-treated NRVMs (Figures 3(a)–3(c)). Because HSF1 activation depends on its translocation to the nucleus to activate the expression of its target genes, nuclear accumulation of HSF1 was examined by immunolocalization using an anti-HSF1 antibody. In control cells, HSF1 was localized to both the cytoplasm and nucleus. In Ang II-treated NRVMs, Ang II stimulated translocation of HSF1 into the nucleus. Choline further enhanced the nuclear localization of HSF1, which was reversed by 4-DAMP (Figure 3(d)). Western blotting revealed that HSF1 expression in the nuclear fraction of the Ang II-treated group was increased relative to the control cells. Compared to the Ang II group, the choline-treated Ang II group had elevated nuclear HSF1 levels, and this effect of choline was blocked by 4-DAMP (Figures 3(e) and 3(f)).

### 3.5. Downregulation of M3AChR Inhibited the Protective Effect of Choline on Ang II-Induced NRVM Apoptosis

**Figure 4:**
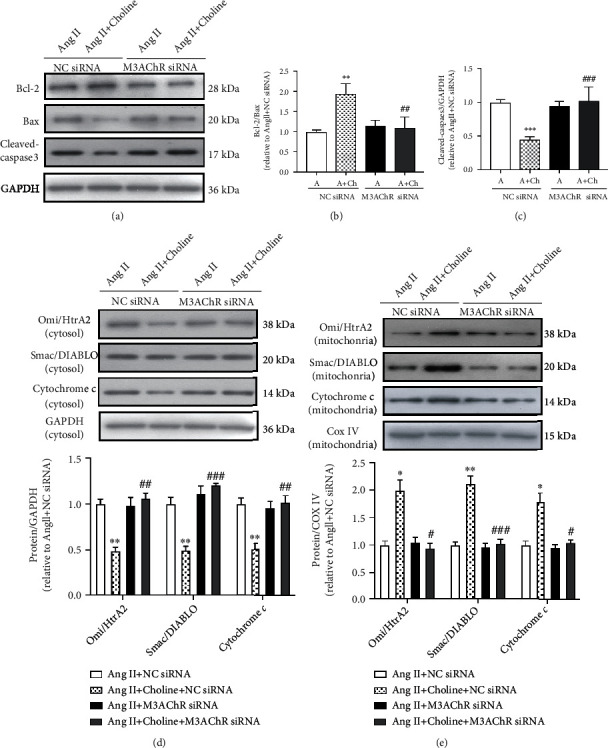
Downregulation of type 3 muscarinic acetylcholine receptors (M3AChR) blocked the protective effect of choline on Ang II-induced cardiomyocyte apoptosis. (a) Choline increased the Bcl-2/Bax ratio and inhibited cleaved-caspase-3 expression; these effects were abolished by M3AChR small interfering ribonucleic acids (siRNAs). (b, c) Quantification of Bcl-2/Bax and cleaved-caspase-3. Data are presented as means ± SD (*n* = 5 independent experiments). (d) Representative image and graph of Omi/HtrA2, Smac/DIABLO, and cytochrome *c* protein levels in the cytosol fraction. Data are presented as means ± SD (*n* = 5 independent experiments). (e) Representative western blot images and data of Omi/HtrA2, Smac/DIABLO, and cytochrome *c* levels in the mitochondrial fraction. Data are presented as means ± SD (*n* = 5 independent experiments). ^∗^*P* < 0.05, ^∗∗^*P* < 0.01, and ^∗∗∗^*P* < 0.001 vs. the negative control (NC) siRNA group; ^#^*P* < 0.05, ^##^*P* < 0.01, and ^###^*P* < 0.001 versus the choline-treated NC siRNA group.

To determine whether M3AChR is associated with choline-mediated suppression of apoptosis during Ang II treatment, M3AChR expression in NRVMs was inhibited by siRNA transfection. Compared to the NC siRNA group, NRVMs transfected with M3AChR siRNA had reduced Bcl-2/Bax ratios and elevated levels of cleaved-caspase-3 in the presence of choline (Figures 4(a)–4(c)).

Next, the relationship between M3AChR and the mitochondrial apoptosis pathway was investigated in cells treated with choline and Ang II. Indeed, when compared to the NC siRNA group, M3AChR knockdown in the presence of choline reduced the expression of cytochrome *c*, Omi/HtrA2, and Smac/DIABLO in the mitochondria, while increasing their expression in the cytosol (Figures 4(d) and 4(e)). These data imply that M3AChR is involved in the protective effect of choline in Ang II-induced cardiomyocyte apoptosis.

**Figure 5:**
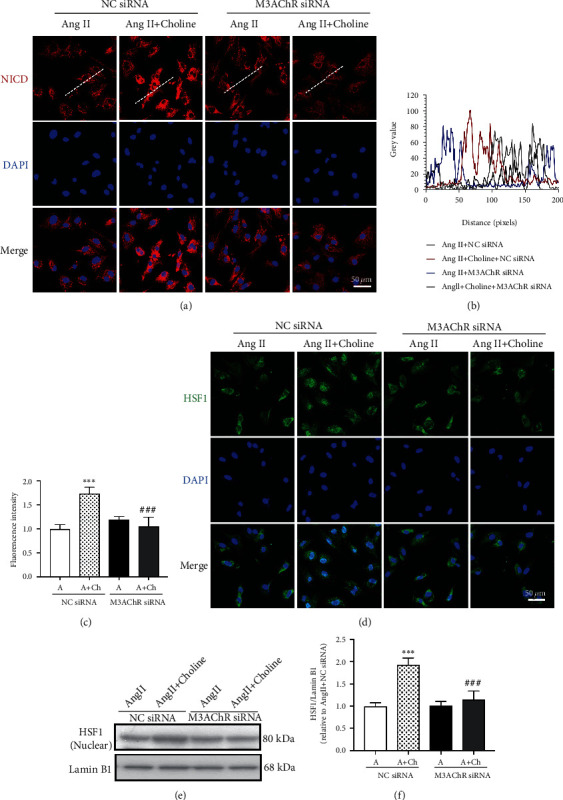
M3AChR siRNA blocked choline-mediated activation of the Notch1/HSF1 pathway. (a–c) The protein levels of NICD (red) in NRVMs were quantified via immunostaining with the indicated antibodies; nuclei were stained with DAPI (blue). Scale bar: 50 *μ*m; *n* = 5 independent experiments. (d) HSF1 immunofluorescence. Results represent five independent experiments. Scale bar: 50 *μ*m. (e, f) Representative images and graph of HSF1 protein levels in the nuclear fraction. Data are presented as means ± SD (*n* = 5 independent experiments). ^∗∗∗^*P* < 0.001 versus the negative control (NC) siRNA group; ^###^*P* < 0.001 versus the choline-treated NC siRNA group.

Next, the role of M3AChR in choline activation of Notch1-HSF1 in Ang II-treated NRVMs was assessed (Figures 5(a)–5(f)). Compared with NRVMs transfected with NC siRNA, those transfected with M3AChR siRNA showed reduced NICD expression and decreased HSF1 levels in the nuclear fraction in the presence of choline.

### 3.6. Downregulation of Notch1 Inhibited the Protective Effect of Choline on Ang II-Induced Apoptosis in NRVMs

**Figure 6:**
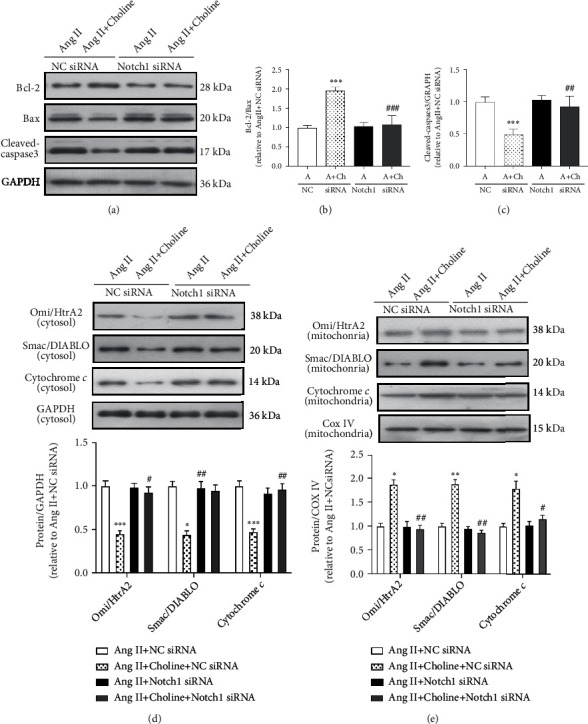
Downregulation of Notch1 blocked the protective effect of choline on Ang II-induced apoptosis. (a) Representative image and graph of Bcl-2, Bax, and cleaved-caspase-3 protein levels. (b, c) Quantification of Bcl-2/Bax and cleaved-caspase-3. Data are presented as means ± SD (*n* = 5 independent experiments). (d) Representative image and graph of Omi/HtrA2, Smac/DIABLO, and cytochrome *c* protein levels in the cytosol fraction. Data are presented as means ± SD (*n* = 5 independent experiments). (e) Representative western blot images and data of Omi/HtrA2, Smac/DIABLO, and cytochrome *c* protein levels in the mitochondrial fraction. Data are presented as means ± SD (*n* = 5 independent experiments). ^∗^*P* < 0.05, ^∗∗^*P* < 0.01, and ^∗∗∗^*P* < 0.001 versus the NC siRNA group; ^#^*P* < 0.05, ^##^*P* < 0.01, and ^###^*P* < 0.001 versus the choline-treated negative control (NC) siRNA group.

To directly demonstrate that the M3AChR/Notch1 pathway is involved in choline-mediated apoptosis modulation, NRVMs were transfected with Notch1 siRNA. These transfected cells had decreased Bcl-2/Bax ratios and higher cleaved-caspase-3 levels relative to the NC siRNA group in the presence of choline (Figures 6(a)–6(c)).

**Figure 7:**
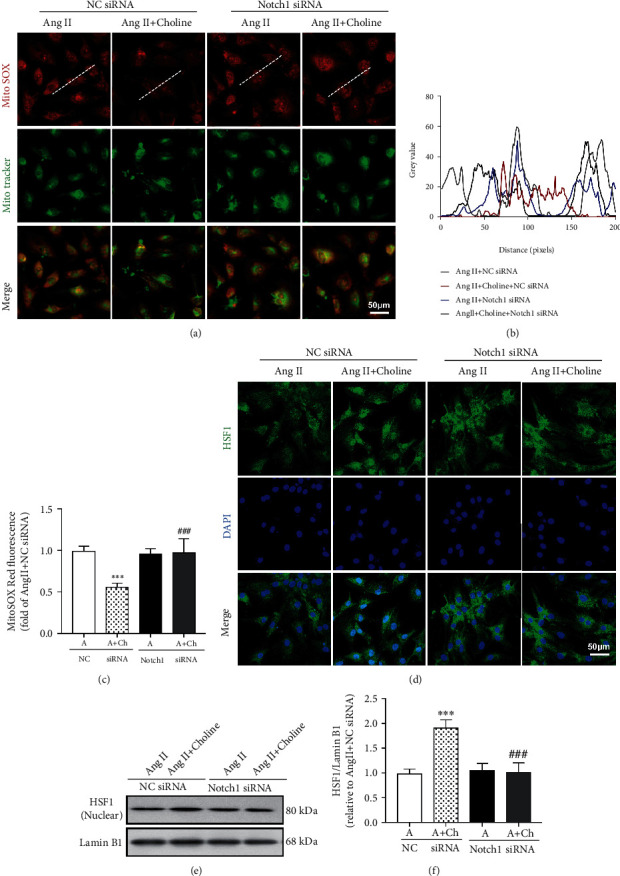
Downregulation of Notch1 blocked the inhibiting effect of choline on Ang II-induced mtROS production and promotion of HSF1 nuclear translocation. (a–c) Representative images and data showing mitochondrial (mt) ROS levels. MitoSOX Red fluorescence was colocalized with MitoTracker Green. Data are presented as means ± SD (*n* = 5 independent experiments). Scale bar: 50 *μ*m. (d) HSF1 immunofluorescence staining. Results represent five independent experiments. Scale bar: 50 *μ*m. (e, f) Representative images and graph of HSF1 protein levels in the nuclear fraction. Data are presented as means ± SD (*n* = 5 independent experiments). ^∗∗∗^*P* < 0.001 versus the negative control (NC) siRNA group; ^###^*P* < 0.001 versus the choline-treated NC siRNA group.

Next, the role of Notch1 in the mitochondrial apoptosis pathway of cells treated with choline and Ang II was determined. Compared with NRVMs transfected with NC siRNA, Notch1 knockdown in the presence of choline reduced the expression of cytochrome *c*, Omi/HtrA2, and Smac/DIABLO in the mitochondria, while increasing their expression in the cytosol (Figures 6(d) and 6(e)). Additionally, in NRVMs with silenced Notch1, mtROS generation and nuclear localization of HSF1 were observed. As shown in Figures 7(a)–7(c), in the presence of Ang II, Notch1 siRNA blocked choline-induced mtROS decreased accumulation. Notch1 silencing also prevented the choline-induced potentiation of Ang II-induced HSF1 nuclear translocation (Figures 7(d)–7(f)). These results indicate that the M3AChR/Notch1 pathway plays a role in the amelioration of Ang II-induced mtROS generation and in the choline-mediated upregulation of HSF1 nuclear translocation.

### 3.7. Choline Attenuated Cardiac Remodeling and Apoptosis in Ang II-Infused Rats

**Figure 8:**
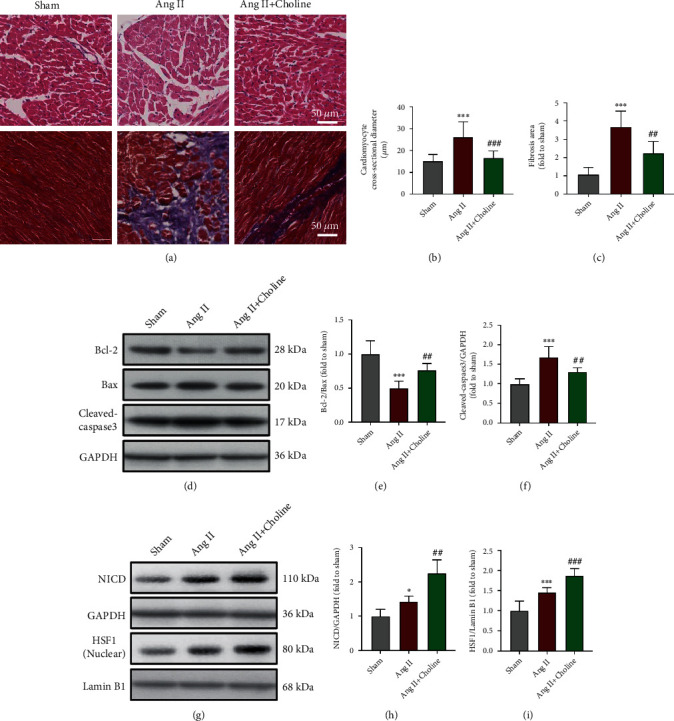
Choline inhibited cardiac remodeling and apoptosis in Ang II-treated rats. (a) Representative left ventricular sections were stained with H&E and Masson's trichrome to assess cardiomyocyte hypertrophy and fibrosis, respectively; scale bars = 50 *μ*m. (b) Cross section of cardiac myocytes in the left ventricle; *n* = 8 rats for each group with three random fields. (c) The level of fibrosis (%) stained by Masson's trichrome; *n* = 8 rats for each group. (d) Representative image and graph of Bcl-2, Bax, and cleaved-caspase-3 protein levels. (e, f) Quantification of Bcl-2/Bax and cleaved-caspase-3; *n* = 8 rats for each group. (g) Representative image and graph of NICD and nuclear HSF1 protein levels. (h, i) Quantification of NICD and nuclear HSF1. Data are presented as means ± SD. ^∗^*P* < 0.05 and ^∗∗∗^*P* < 0.001 versus sham; ^##^*P* < 0.01 and ^###^*P* < 0.001 versus the Ang II group.

To evaluate the specific effects of choline on cardiac remodeling and apoptosis, rats were subjected to Ang II infusion to induce cardiac remodeling and apoptosis. Histopathological studies revealed a larger cardiomyocyte diameter and greater interstitial fibrosis in the Ang II group compared with the sham group. These effects were significantly attenuated in rats treated with choline (Figures 8(a)–8(c)). Western blotting analyses revealed that the Bcl-2/Bax ratios were decreased, but that of cleaved-caspase-3 was increased in the Ang II-treated rats. Choline significantly increased the ratio of Bcl-2/Bax and decreased cleaved-caspase-3 expression (Figures 8(d)–8(f)). The Ang II-infused rats also showed higher levels of NICD and nuclear HSF1 than the sham group (Figures 8(g)–8(i)). Compared to the Ang II group, the NICD and nuclear HSF1 expression levels were further increased in the choline-treated Ang II group. These results suggest that choline-mediated prevention of Ang II-induced cardiac remodeling and apoptosis is associated with activation of the Notch1/HSF1 pathway.

## 4. Discussion

**Figure 9:**
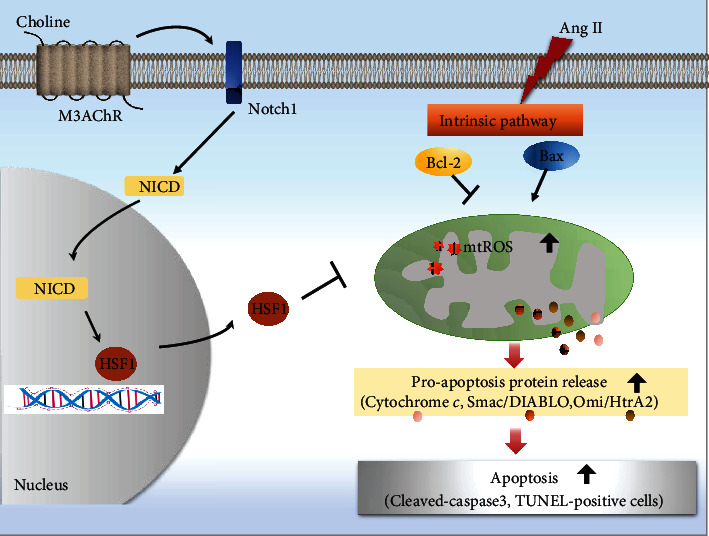
Choline likely attenuates Ang II-induced cardiomyocyte apoptosis by inhibiting mtROS generation and the release of proapoptotic proteins from the mitochondria via the M3AChR and Notch1/HSF1 pathway. Ang II: angiotensin II; M3AChR: type 3 muscarinic acetylcholine receptor; HSF1: heat shock transcription factor 1; Omi/HtrA2: Omi/high-temperature requirement protein A2; Smac/DIABLO: second mitochondrial activator of caspases/direct inhibitor of apoptosis-binding protein with low P; mtROS: mitochondrial reactive oxygen species.

Ang II activation in the heart has been associated with the development of cardiac hypertrophy and apoptosis, which are thought to be a common pathological basis of myocardial infarction, dilated cardiomyopathy, and early-stage HF. This study evaluated the effects of choline on oxidative stress damage and the mitochondrial death pathway in Ang II-treated NRVMs. The findings of this study can be summarized as follows: (1) in cultured NRVMs, choline increased NICD expression and promoted HSF1 nuclear translocation, thereby inhibiting the excessive production of mtROS. Accordingly, choline, likely via M3AChRs, attenuated Ang II-induced release of cytochrome *c*, Omi/HtrA2, and Smac/DIABLO from the mitochondria, thereby alleviating Ang II-induced cardiomyocyte hypertrophy and apoptosis. (2) M3AChR siRNA was observed to reduce choline-mediated upregulation of NICD and HSF1 translocation, as well as blocking choline-mediated inhibition of the mitochondrial apoptosis pathway. These findings support the hypothesis that M3AChR activation is responsible for the cardioprotective effects of choline. (3) Notch1 siRNA diminished the choline-mediated promotion of HSF1 nuclear translocation, as well as inhibition of mtROS and mitochondrial apoptosis pathway, indicating that the Notch1/HSF1 pathway is a key regulator of cardiomyocyte apoptosis. (4) Choline attenuated cardiac remodeling and apoptosis in Ang II rats in a manner related to activation of the Notch1/HSF1 pathway, consistent with the *in vitro* findings. Taken together, our results indicate that Notch1/HSF1 regulates mtROS generation and the intrinsic apoptotic pathway in Ang II-treated NRVMs. Our results also demonstrate that choline counteracts mtROS production and cardiomyocyte apoptosis in part by activating M3AChRs and the Notch1/HSF1 signaling pathway (Figure 9).

An elevated level of Ang II, a vessel contractor molecule, in the heart is associated with an increased rate of myocardial apoptosis [26, 27]. Many studies have demonstrated that inhibition of apoptosis is cardioprotective and can prevent the development of HF [9]. Pharmacological intervention is one of the several strategies used to reduce apoptosis during cardiac injury [27]. The results here show that Ang II-treated NRVMs undergoing apoptosis are characterized by increased expression and numbers of cleaved-caspase-3 and TUNEL-positive cells, respectively, the effects of which were significantly inhibited by choline. Apoptosis is a biological process, which, in mammalian cells, is regulated by two major pathways. The intrinsic or mitochondrial pathway is regulated by Bcl-2 proteins and is characterized by ROS generation, depolarization of mitochondrial membrane potential, and the release of apoptogenic factors such as cytochrome *c* from the mitochondria to the cytoplasm [28, 29]. In this study, Ang II activated the intrinsic mitochondrial apoptotic pathway, as evidenced by decreased Bcl-2/Bax ratios and enhanced mitochondrial release of cytochrome *c*, Omi/HtrA2, and Smac/DIABLO. On exposure to a pathological insult, Omi/HtrA2 and Smac/DIABLO are translocated from the mitochondria to the cytosol where they promote apoptosis via a protease activity-dependent and caspase-mediated pathway. Following their release, Smac/DIABLO and Omi/HtrA2 bind to inhibitors of apoptotic proteins (IAPs), blocking their ability to inhibit caspases and consequently promoting apoptosis [30, 31]. Notably, Ang II-induced activation of the intrinsic apoptotic pathway was reversed by choline. These findings support a correlation between Ang II treatment and mitochondrion-dependent apoptotic pathway.

Notch1 signaling has been reported to be important for proper myocardial function and response to injury, especially in myocardial ischemia I/R injury [32], myocardial infarction [33], and cardiac hypertrophy [34]. Previous studies have found that Notch1 knockdown significantly aggravated myocardial I/R injury, as evidenced by an enlarged infarct size, depressed cardiac function, and increased myocardial apoptosis [35]. Our present data demonstrate that choline increased the NICD expression and promoted HSF1 nuclear translocation, thereby attenuating Ang II-induced cardiomyocyte hypertrophy and apoptosis. Furthermore, Notch1 siRNA inhibited choline-mediated antiapoptotic effects. The result further confirmed the cardioprotective effects of Notch1 activation. Notch1 also plays a critical role in ROS production, and an excessive concentration of intracellular ROS contributes to the activation of the apoptotic signaling pathway [10]. It has been previously demonstrated that Notch1 activation inhibited ROS production in hepatic and myocardial I/R injury [10, 13]. Cai et al. reported that the Notch1 pathway protects against burn-induced myocardial injury by repressing ROS production and activating the expression of MnSOD [36]. Similarly, the data here indicate that the mitochondrial ROS level was significantly elevated by Ang II administration, which was reversed by choline. Moreover, siRNA-mediated downregulation of Notch1 led to increases in mtROS levels and cardiomyocyte apoptosis, implying that the cardioprotective effects of the Notch1 pathway are involved in the regulation of mtROS levels.

In addition to regulating mtROS production, Notch1 has been reported to regulate the cellular stress response machinery in central T cell acute lymphoblastic leukemia by inducing the expression of HSF1 and its downstream effectors [37]. A recent study demonstrated that Notch1 signaling promoted HSF1 activation, which in turn inhibited NLRP3 function and hepatocellular apoptosis, leading to the alleviation of I/R-induced liver injury [13]. Consistent with these results, we demonstrate for the first time that choline increased NICD expression and promoted HSF1 nuclear translocation in cardiomyocytes, which then attenuated Ang II-induced cardiomyocyte hypertrophy and apoptosis. Notably, Notch1 siRNA abolished the choline-mediated promotion of HSF1 nuclear translocation and cardiomyocyte apoptosis, indicating that choline-mediated activation of the Notch1/HSF1 pathway is a key regulator of cardiomyocyte apoptosis.

HSF1 is considered a cardioprotective factor that controls the expression of heat shock protein during the stress response in cardiomyocytes [38–40]. Previous studies have reported that HSF1 deficiency accelerated the transition from pressure overload-induced cardiac hypertrophy to HF [41]. A recent study demonstrated that HSF1 functioned as a key defender against palmitic acid-induced ferroptosis in cardiomyocytes [42]. In contrast, Huang et al. found that Ang II treatment promoted HSF1 acetylation, which induced insulin-like growth factor receptor II expression and eventually resulted in cardiac hypertrophy and apoptosis of H9c2 cells [11]. Liu et al. showed that HSF1 acted as a transcriptional factor that induced Omi/HtrA2 expression and caspase-9 apoptosis in aged cardiomyocytes, while also decreasing cardiac function reserve [43]. Interestingly, we found that Ang II stimulation promoted HSF1 nuclear translocation and that choline treatment resulted in higher activation of HSF1 nuclear translocation and less apoptosis, indicating that choline-mediated activation of HSF1 nuclear translocation is beneficial. Future studies should investigate the involvement of posttranslational modifications of HSF1 to explain its role in multiple models of heart injury.

Muscarinic acetylcholine receptors mediate diverse physiological and pathological functions. Previous studies have showed that acetylcholine-induced autophagy attenuated apoptosis in H9c2 cells, the effects of which were blunted by muscarinic receptor antagonist atropine, suggesting that the muscarinic receptor plays a pivotal role in cardioprotection [44]. At present, five muscarinic acetylcholine receptor subtypes (M1–M5) have been identified and their importance as novel targets in cardiovascular disease is increasingly being appreciated [45]. Emerging evidence argues for a critical role of type 2 muscarinic acetylcholine receptor (M2AChR) and M3AChR in the modulation of cardiac diseases. Li et al. demonstrated that acetylcholine inhibited tumor necrosis factor-*α* production in cardiomyocytes and that M2AChR antagonists abolished the cardioprotective effects offered by acetylcholine, further supporting the functional role of M2AChR [46]. In addition, pharmacological activation of M3AChR by choline was found to be cardioprotective in myocardial infarction [47], arrhythmias [48, 49], and cardiac hypertrophy [50]. However, the role of M3AChR activation by choline in oxidative stress and cardiomyocyte apoptosis is yet to be determined. In this study, choline was found to activate the Notch1 signaling pathway, promote HSF1 nuclear translocation, and suppress Ang II-induced increases in mtROS levels and release of proapoptosis proteins (i.e., cytochrome *c*, Omi/HtrA2, and Smac/DIABLO) from the mitochondria, thereby alleviating cardiomyocyte apoptosis. Notably, 4-DAMP or M3AChR siRNA abolished these effects of choline, indicating that M3AChR is responsible for the cardioprotective effect of choline.

One limitation of this study is that the doses of Ang II used are higher than the levels of Ang II that are normally present in plasma. Pharmacological doses of Ang II are usually used in Ang II signaling experiments and may or may not mimic physiological conditions [51]. Most *in vitro* studies use a single high dose of Ang II (10^−6^ or 10^−7^ mol/L). In this study, 10^−6^ mol/L Ang II had marked effects on cardiomyocyte apoptosis; thus, 10^−6^ mol/L Ang II was used for subsequent experiments. This dose is consistent with the concentrations used in previous studies [17].

Taken together, our results demonstrate for the first time that choline attenuates Ang II-induced oxidative stress and apoptosis, likely via the M3AChR and Notch1/HSF1 signaling pathway. Our findings also imply that Notch1 is a novel regulator of cardiomyocyte apoptosis. These findings provide important insights into the molecular mechanisms underlying the cardioprotective effect of choline and may help inform the development of novel therapeutic targets for treating cardiac damage and HF with a focus on cardiomyocyte apoptosis.

## Data Availability

The data used to support the findings of this study are available from the corresponding author upon request.

## Supplementary Materials

Supplementary Materials Supplementary Figure S1: protein expression levels of Bcl-2, Bax, and cleaved-caspase-3 of different concentrations and times of Ang II in primary cardiomyocytes.
