# Supplementary material for: Activation of the M3AChR and Notch1/HSF1 Signaling Pathway by Choline Alleviates Angiotensin II-Induced Cardiomyocyte Apoptosis
Source: Oxid Med Cell Longev. 2021 Aug 30;2021:9979706. doi: 10.1155/2021/9979706 (PMC8423579; doi:10.1155/2021/9979706)
Supplement: Supplementary Materials — Supplementary Figure S1: protein expression levels of Bcl-2, Bax, and cleaved-caspase-3 of different concentrations and times of Ang II in primary cardiomyocytes. [file 9979706.f1.docx]

**Supplemental figures and legends**

**Title**

Activation of the M3AChR and Notch1/HSF1 signaling pathway by choline alleviates angiotensin II-induced cardiomyocyte apoptosis

**Authors**

Man Xu^1^, Xue-Yuan Bi^2^*, Xiao-Rong Xue^1^*, Xing-Zhu Lu^3^, Qiong-Ge Li^1^, Qiang Jian^1^, Jian-Yong Sun^4^

**Affiliations**

1. Department of Clinical Pharmacy, Xi’an People’s Hospital (Xi'an Fourth Hospital), Xi'an 710004, Shaanxi Province, China

2. Department of pharmacy, Hong Hui Hospital, Xi'an Jiaotong University, Xi'an 710054, Shaanxi Province, China

3. Department of pharmacy, Second Affiliated Hospital of Xi’an Jiaotong University Medical School, No.157, West Fifth Road, Xi’an, Shaanxi, 710004, China

4. Department of Thoracic Surgery, Tangdu Hospital, Air Force Medical University, Xi'an 710004, Shaanxi Province, China

***Corresponding author****:**

Xue-Yuan Bi, PhD, Department of pharmacy, Hong Hui Hospital, Xi'an Jiaotong University, Shaanxi 710054, China.

Tel: +86-29-85220671; Fax: +86-29-85220671; E-mail: bixueyuan@mail.xjtu.edu.cn

Xiao-Rong Xue, MS, Department of Clinical Pharmacy, Xi'an People’s Hospital (Xi'an Fourth Hospital), Xi'an 710004, Shaanxi Province, China.

Tel: +86-29-87480635; Fax: +86-29-87480635; E-mail: [xuexiaorong2006@163.com](mailto:xuexiaorong2006@163.com)

Xue-Yuan Bi and Xiao-Rong Xue contributed equally to this work.

**
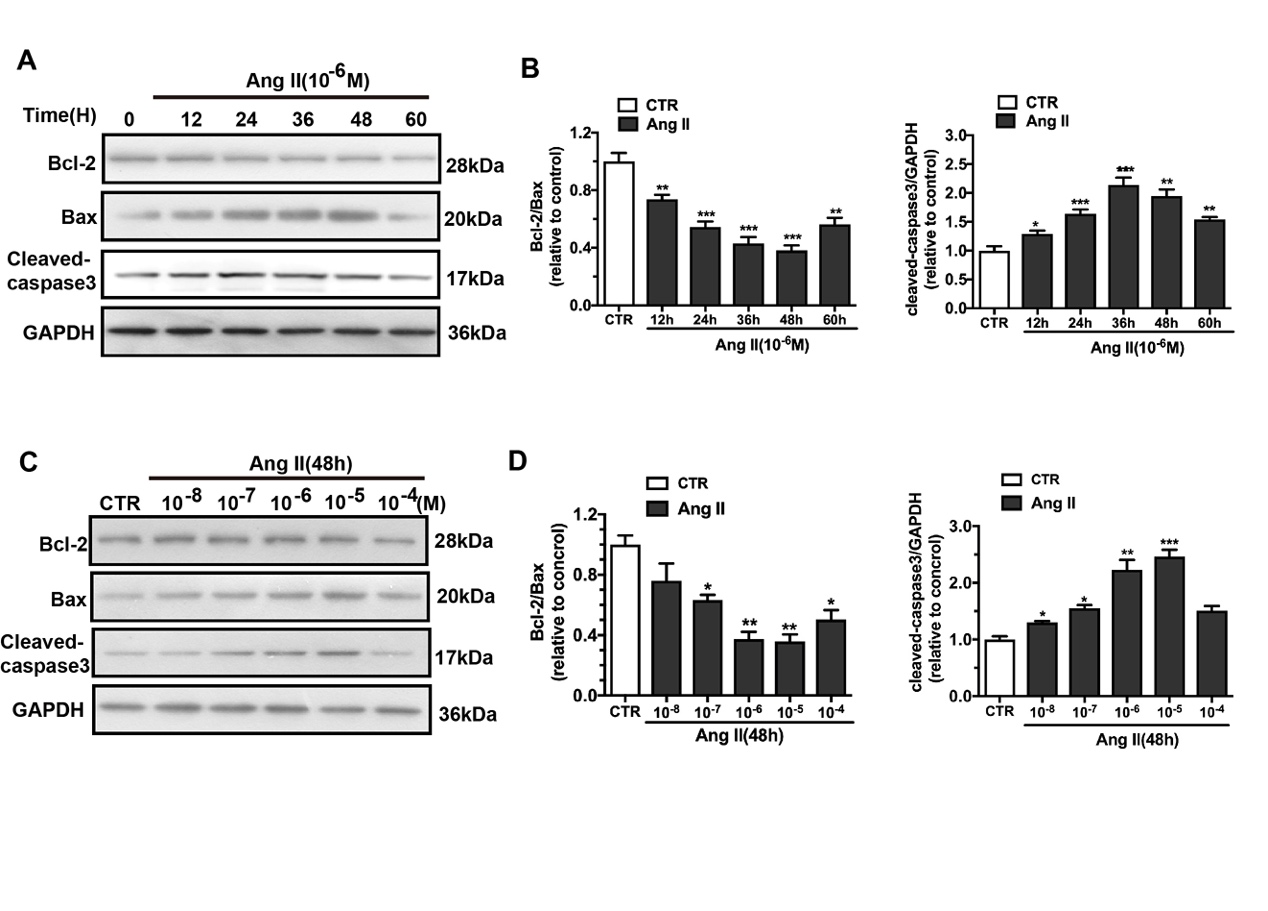
**

**Figure S1.** **Angiotensin II (Ang II) treatment induced apoptosis of neonatal rat ventricular myocytes (NRVMs).** (A, B) NRVM lysates at different time courses following Ang II treatment was immunoblotted with antibodies against Bcl-2, Bax, and cleaved-caspase-3, *n* = 5 independent experiments. (C, D) Ang II decreased the Bcl-2/Bax ratio and dose-dependently increased the expression of cleaved-caspase-3; *n* = 5 independent experiments. Data are presented as means ± SD. **P* < 0.05, ***P* < 0.01, ****P* < 0.001 versus control (CTR).
